# Histology of Non-Melanoma Skin Cancers: An Update

**DOI:** 10.3390/biomedicines5040071

**Published:** 2017-12-20

**Authors:** Giovanni Paolino, Michele Donati, Dario Didona, Santo Raffaele Mercuri, Carmen Cantisani

**Affiliations:** 1 Dermatology and Cosmetology, IRCCS San Raffaele Hospital, 20132 Milan, Italy; paolgio@libero.it (G.P.); mercuri.santoraffaele@hsr.it (S.R.M.); 2 Department of Dermatology, Dipartimento di Medicina Interna e Specialità Mediche, La Sapienza University of Rome, 00161 Rome, Italy; 3 Department of Pathology, Università Campus Biomedico, 00161 Rome, Italy; micheledonati25@gmail.com; 4 First Division of Dermatology, IRCCS Istituto Dermopatico dell’Immacolata, 00161 Rome, Italy; dario.didona@gmail.com; 5 Unità Operativa Complessa of Dermatology, Policlinico Umberto I, Sapienza University of Rome, Viale del Policlinico 155, 00161 Rome, Italy

**Keywords:** non-melanoma skin cancers, basal cell carcinoma, squamous cell carcinoma, diagnosis, pathology

## Abstract

Non-melanoma skin cancer (NMSC) is the most frequently diagnosed cancer in humans. Several different non-melanoma skin cancers have been reported in the literature, with several histologic variants that frequently cause important differential diagnoses with other cutaneous tumors basal cell carcinoma (BCC) is the most common malignant skin tumor, with different histologic variants that are associated with a greater or less aggressive behavior and that usually may be confused with other primitive skin tumors. Actinic keratosis, Bowen’s disease, keratoacanthoma, and invasive squamous cell carcinoma (SCC) correspond to the other line of NMSC, that may have only local tumoral behavior, easy to treat and with local management (as in the case of actinic keratosis (AK), Bowen’s disease, and keratoacanthoma) or a more aggressive behavior with a potential metastatic spread, as in case of invasive SCC. Therefore, histopathology serves as the gold standard during daily clinical practice, in order to improve the therapeutical approaches to patients with NMSC and to understand the distinct histopathological features of NMSC. Here, we reported the main pathological features of different non-melanoma skin cancers.

## 1. Introduction

Skin cancer is a worldwide, emerging clinical need in the elderly white population, with a steady increase in incidence rates, morbidity, and related medical costs [1]. Skin cancer is a heterogeneous group of cancers comprising cutaneous melanoma and non-melanoma skin cancers (NMSC), which predominantly affects patients older than 65 years of age [1]. Under the umbrella of NMSC are all the non-melanoma malignant neoplasms affecting the skin [1]. However, especially epidemiologically, the term NMSC practically refers to keratinocyte carcinomas, namely basal cell carcinoma (BCC), squamous cell carcinoma (SCC), and actinic keratosis (AK). Because they account for the 99% of the tumors in this group, we will focus on these types of NMSC.

## 2. Discussion

### 2.1. Basal Cell Carcinoma and Its Histological Variants

#### 2.1.1. General Considerations

BCC is a cutaneous malignant proliferation, which derives from basaloid cells and accounts for 50% of all cancers in the United States [1,2,3]. It has been postulated that BCC derives from the basal cell layer and outer root sheath of the hair follicle; these cells are pluripotent epithelial cells [1]. In addition, the expression of CD10 emphasizes the follicular derivation of these epithelial cells; more specifically, the absence of cytokeratin 15 in BCC suggests that these pluripotent cells arise from the bulge region of the hair follicle [1,4]. Strong sun-exposure, immunosuppression, beta-Human Papilloma Virus (HPV), and human immunodeficiency virus (HIV) increase the risk of BCC in the general population [1].

#### 2.1.2. Main Histologic Variants

Several different variants of BCC have been reported, which show variable outcomes and prognoses [1]. In this paragraph, we analyze the different BCC histologic patterns, according to the different types that clinicians could find in the daily clinical practice (Table 1).

**Figure 1:**
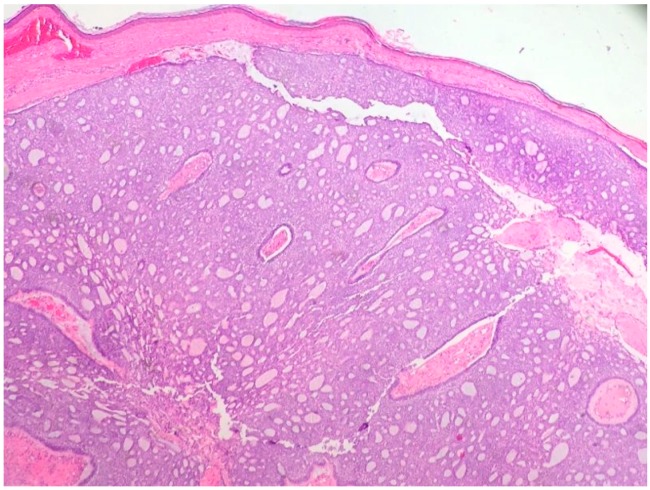
Nodular basal cell carcinoma (hematoxylin and eosin, 20×).

**Figure 2:**
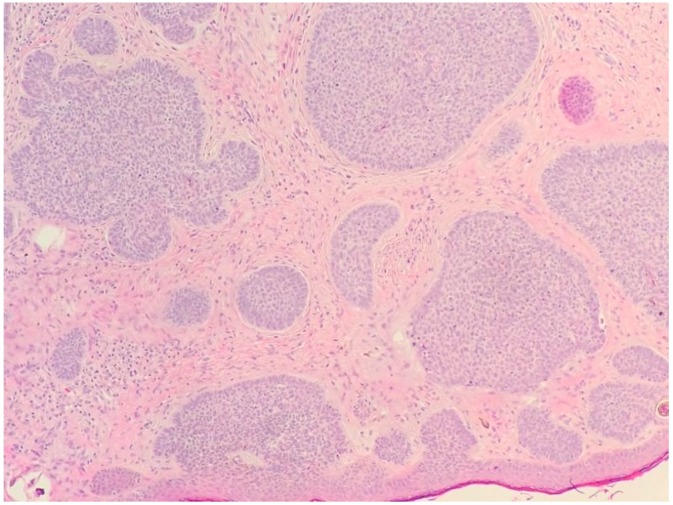
Nodular basal cell carcinoma (hematoxylin and eosin, 20×).

The nodular type accounts for 50% of all BCC [1] (Figure 1 and Figure 2). Nodular BCC (NBCC) is characterized by aggregates of basaloid cells with well-defined borders, showing a peripheral palisading of cells and a typical cleft [1,5,6]. Central necrosis with eosinophilic, granular features may be also present, as well as mucin [1,2,3]. The heavy aggregates of mucin determine a cystic structure; calcification may be also present, especially in long-standing lesions [1]. Mitotic activity is usually not so evident, but a high mitotic rate may be present in more aggressive lesions [1]. Adenoidal BCC can be classified as a variant of NBCC, characterized by basaloid cells with a reticulated configuration extending into the dermis [1,2,3,7,8]. The main differential diagnosis of adenoid BCC is adenoid cystic carcinoma and sometimes it is very difficult to differentiate the nature of the basaloid tumor cells, since in adenoid cystic carcinoma there are usually basaloid tumor cells with hyperchromatic nucleus arranged in a cribriform pattern undergoing dedifferentiation within a collagenous stroma. However, the lack of basalioid cells disposed in peripheral palisades in an adenoid cystic lesion without connection to the overlying epidermis and the absence of artefactual clefts between the tumor and the stroma help the distinction between an adenoid cystic carcinoma and BCC [2,3].

NBCC with granular features and large cytoplasmic inclusions has been defined as granular cell basal cell carcinoma, characterized by an eosinophilic and granular cytoplasm [1,9]. Interestingly, it has been reported that a percentage of BCC may show clear cells, which may be present at the periphery of the lesion, as well as in the center [1]. These cells are round or polyhedral with pale, eosinophilic, vacuolated, or finely granular cytoplasm [9]. Basal cell epithelioma, another variant of NBCC, is characterized by monster cells, which are enlarged, mononuclear, and/or multinucleated [1,10,11]. BCC with outer hair follicle sheath differentiation is another variant, characterized by infundibular structures of the outer sheath [1].

Micro-nodular BCC is characterized by a plaque-like shape, showing an increase incidence of recurrence [1]. Pathologically, it is similar to NBCC, but it is smaller and forms micro-nodules of basaloid cells, approximately the size of the hair bulbs, with minimal palisading [1,12]. The surrounding stroma is myxoid [1].

Superficial BCC is another common variant of BCC [1]. It is characterized by nests of basaloid cells that extend from the epidermis, with neoplastic cells that resemble primordial germ cells. Peripheral palisading is usually prominent and tumor islands show a well demarcated border [1].

The infiltrative variant of BCC is a continuum between the nodular and morpheiform variant [11]. This variant is characterized by the presence of atypical basaloid cells in nodules that show different sizes, with a surrounding mucinous stroma [1,13]. The invasion of the sub-cutis and of the muscle, as well as of the adnexal structures, may be present. According to these features, this variant of BCC is more aggressive and more difficult to eradicate [1,13]. Another aggressive variant is morpheiphorm BCC, also known as fibrosing BCC [1,14]. This variant, also called sclerosing BCC, is mainly characterized by the presence of thin and elongated tumor islands [14,15]. The peripheral palisading is absent and the stroma retraction is also infrequent [14,15]. The mitotic activity is usually detectable, although not so marked. Morpheiform BCC may involve the fat and cutaneous annexes [1,15].

**Figure 3:**
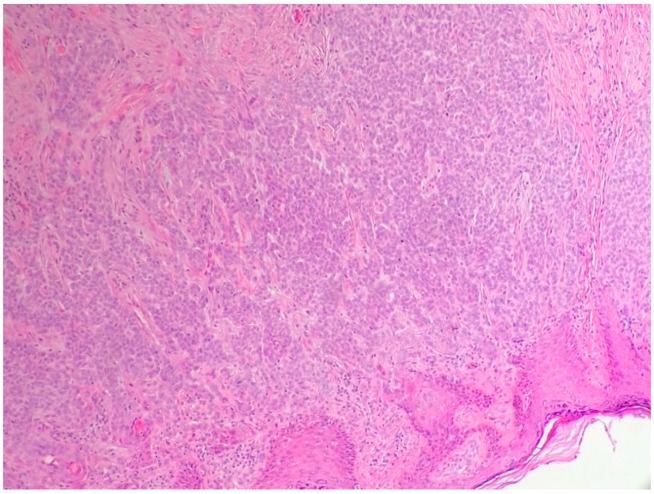
Metatypical basal cell carcinoma (hematoxylin and eosin, 20×).

**Figure 4:**
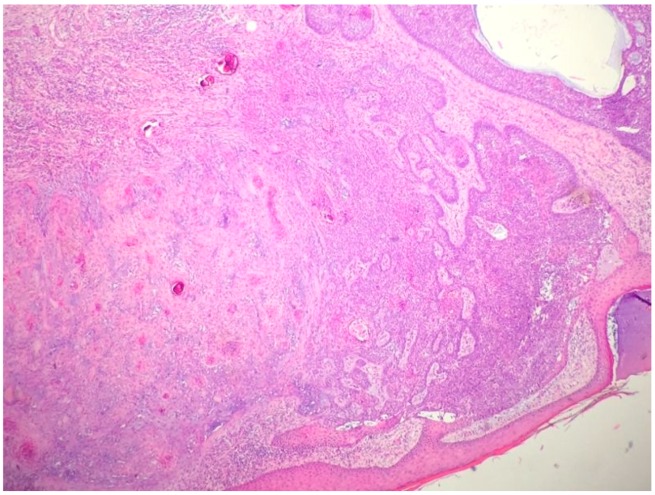
Basosquamous carcinoma (hematoxylin and eosin, 20×).

Metatypical carcinoma (BCC with squamous differentiation) is a BCC with features of nodular BCC and SCC [12]. This variant is characterized by basaloid cells with variable eosinophilic features, prominent mitotic activity, and numerous apoptotic cells [16,17] (Figure 3). Peripheral palisading is not always present. Metatypical carcinoma is one of the most aggressive subtypes, with a higher incidence of peri-neural and lymphatic spread [1,16,17]. Contrariwise, the term baso-squamous carcinoma should be used in the case of tumors with contiguous areas of BCC and SCC [1] (Figure 4). In this case, the typical features of both tumors are present [18].

The main histologic differential diagnoses of BCC are trichoblastoma, merkel cell carcinoma, desmoplastic trichoepithelioma, and microcystic adnexal carcinoma [1] (Table 1).

Specifically, trichlobastoma shows a peri-tumoral stroma that is dense and circumscribed, absence of clefting between tumor cells and stroma (while it is present in BCC), rudimentary hair germs with epithelial strands enclosing primitive mesenchyme, also known as ‘papillary mesenchymal bodies’, features that are absent in BCC. Finally, the peripheral palisading is more prominent in BCC, although it can also be found also in trichoblastoma [2,3] (Table 1).

**Figure 5:**
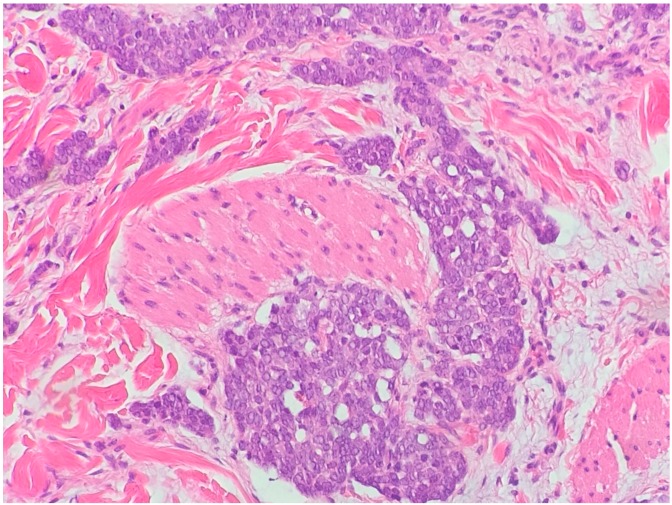
Typical small-to-medium-sized basophilic tumoral cells in a Merkel cell carcinoma. (hematoxilin and eosin, 30×).

Merkel cell carcinoma is characterized by deeply basophilic tumoral cells with small-to-medium-sized cells, sparse cytoplasm, with dense round nuclei (Figure 5). Tumor cells are arranged in a diffuse, trabecular, and/or nested pattern, usually involving the subcutis. Contrariwise, BCC shows a more asymmetrical architecture, palisading of peripheral tumor cells, and a typical cleft between tumor cells and stroma (absent in Merkel cell carcinoma) [2,3] (Table 1).

**Figure 6:**
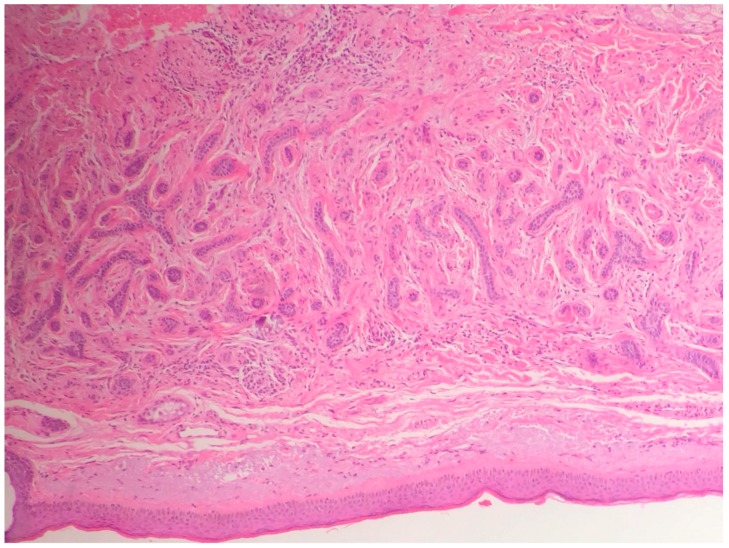
Sometimes the differential diagnosis between a morpheaform BCC and a desmoplastic tricoepithelioma. Rims of collagen bundles surrounding basaloid cells without peripheral palisading and without the typical cleft of BCC, in a desmoplastic tricoepithelioma (hematoxilin and eosin, 10×).

Clinical and histopathological differentiation between morpheaform BCC and trichoepithelioma (above all desmoplastic tricoepithelioma) is usually a frequent problem (Figure 6). BCC is often an asymmetrical lesion, while trichoepithelioma is symmetrical, horn cysts are more frequent in trichoepithelioma, the ulceration is frequent in BCC and rare in trichoepithelioma [2,3]. Besides, rims of collagen bundles, calcification, follicular/sebaceous/infundibular differentiation, and cut artefacts are usually present in trichoepithelioma, while they are not present in BCC. Morpheaform BCC express usually epithelial cell adhesion molecule (EPCAM) (BerEP4), cytokeratin 6 (CK6, that is negative in tricoepithelioma), Ki-67 (negative in trichoepithelioma), and androgen receptor (AR) [19,20,21]. Finally, CD10+ is positive in BCC tumor cells and positive in the stroma in tricoepithelioma. cytokeratin 20 (CK20) is present in tricoepithelioma, as well as p75 and PHLDA1 are positive mainly in desmoplastic tricoepithelioma [2,3,21] (Table 1).

Microcystic adnexal carcinoma can be also confused with a BCC, positive pattern for the expression of carcinoembryonic antigen and epithelial membrane antigen, demonstrating follicular and eccrine differentiation and negative stain for Ber-Ep4 [2,3]. Histologically microcystic adnexal carcinoma is characterized by cords and nests of bland keratinocytes, keratin cysts, presence of a ductal differentiation, and a dense collagenous stroma [2,3] (Table 1).

### 2.2. The Histologic Features of Actinic Keratosis, Bowen’s Disease, and Squamous Cell Carcinoma

#### 2.2.1. General Considerations

SCC accounts approximately for 20% of all cutaneous malignancies. In addition, SCC accounts for the most of NMSC-related metastatic disease and death [1]. Furthermore, SCC is reported to be within the top five most costly cancers in the United States [1]. In the future, SCC incidence will drastically increase, due to an increment in sun exposure, intensifying UV exposure, advancing age of the US population, enhanced public awareness of skin cancer, and more frequent skin examinations by physicians [1]. Cigarette smoking, therapeutic UV light exposure (including PUVA therapy and tanning bed use), HPV infection (types 16, 31 and 34), arsenic ingestion, polycyclic aromatic hydrocarbon exposure, immunosuppression, diets high in fat and meat, preexisting chronic dermatoses, and ulcer or sinus tract formation have been reported as risk factors [18,19,20,21,22,23]. Cutaneous conditions associated with the development of SCC include porokeratosis, lupus erythematosus, lichen planus, lichen sclerosus, epidermolysis bullosa, erythema ab igne, acrodermatitis chronic atrophicans, ichthyosis, dissecting cellulitis of the scalp, chromoblastomycosis, port wine stain, arteriovenous malformation, nevus sebaceus, linear epidermal nevus, granuloma inguinale, fibroepithelial polyps, acne conglobata, necrobiosis lipoidica, verruciform xanthoma, and lymphogranuloma venereum [23,24,25,26,27,28].

Deeper skin invasion is associated with increased metastatic disease and recurrence rates [21]. Lesions greater than 2 cm in diameter are twice as likely to recur and three times as likely to metastasize [19]. Tumor thickness may be measured in millimeters [21]. Peri-neural invasion may be present, and it reduces the five-year survival rate [1,25]. In general terms, these tumors are larger at presentation (>2 cm in diameter), less differentiated, and are associated with lymphadenopathy [17].

Generally, metastatic disease is uncommon [1,21]. The metastatic overall rate is about 2%, but it is between 10% and 15% for lesions on the lips and ears [1,19]. Eighty-five percent of metastases affect only the lymph nodes, but involvement of lungs, liver, bones, brain, and mediastinum may also occur [1,21].

#### 2.2.2. Actinic Keratosis

Among non-melanoma skin cancer (NMSC), squamous cell carcinoma (SCC) accounts for the majority of NMSC-related metastases and death. A wide diversity of SCC subtypes exist, several of which are associated with markedly more aggressive behavior and other with lower aggressive behavior (Table 2). Hoverer, below we report the main histologic variant that usually we encounter during the daily clinical practice: actinic keratosis, Bowen’s disease, invasive SCC, and keratoacanthoma (Table 2).

**Figure 7:**
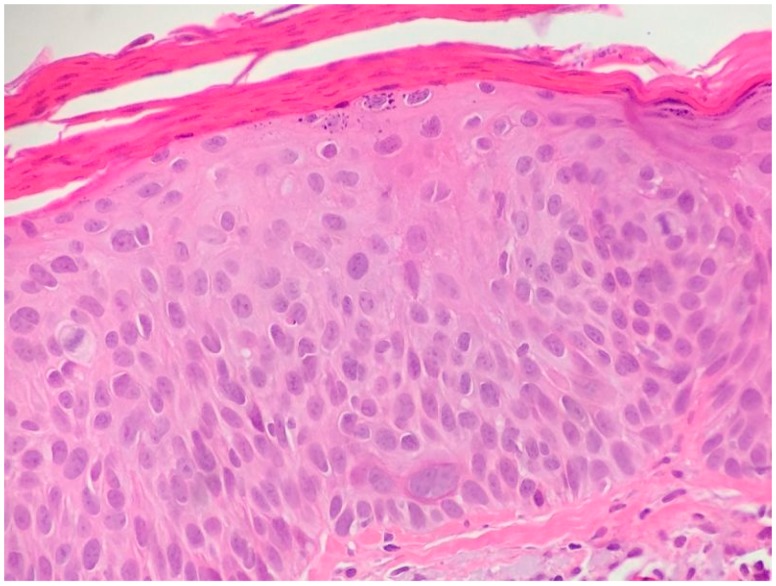
Actinic keratosis with Bowenoid features (hematoxylin and eosin, 40×).

Actinic keratosis (AK) can be considered as an early SCC, rather than a pre-malignant lesion [1,21]. AK could potentially evolve into invasive tumor [1,21,28]. Pathologically, AK is characterized by a horizontal alteration of parakeratotic and orthokeratotic hyperkeratosis, with an atrophic or acanthotic epidermis (Figure 7) [21]. Neoplastic keratinocytes in the basal layer show increased cellularity, nuclear pleomorphism, and scattered mitoses [21]. A superficial lichenoid or perivascular lymphocytic infiltrate is often associated with plasma cells [21]. In the papillary dermis, elastosis is often present [21]. The main histologic variants of AK are the acantholitic and the pigmented ones. Acantholitic AK is characterized by hyperkeratosis, acanthosis, and suprabasal acantholysis characterized by atypical basal keratinocytes. Acantholytic AK can be misdiagnosed for other acantholytic diseases as Grover disease, Darier disease, warty dyskeratoma, as well as seborrheic keratosis with acantholysis. Pigmented AK is characterized by melanin pigment in the keratinocytes and melanophages; specifically, the melanin is focally increased in basal keratinocytes. However, a slight melanocytic proliferation may be present [2,3]. The main differential diagnosis are Bowen’s disease (atypia of keratinocytes at every layer of the epidermis) and flat seborrheic keratosis, that is characterized by acanthosis and papillomatosis with sharp usually flat base and distinct lateral margins, intraepithelial horn pseudo-cysts, and monotonous basaloid tumor cells without atypia [21].

#### 2.2.3. Main Histologic Variant and Differential Diagnoses of NMSC with Squamous Differentiation

Bowen’s disease can be defined as an intra-epidermal carcinoma with atypia of keratinocytes at every layer of the epidermis (squamous cell carcinoma in situ) [21]. Pathologically, it is characterized by parakeratosis and by bulbous rete ridges in the epidermis [21]. In addition, the entire epidermis shows disordered maturation with atypical keratinocytes, individual cell keratinization, pleomorphic nuclei, atypical mitoses, multi-nucleated tumor-cells, and an intact basal layer [21]. A lymphocytic perivascular or lichenoid infiltrate is present, often with mixed plasma cells [21]. The main histological differential diagnoses are erytroplasia of Queyrat (identical to Bowen’s disease but on mucosa), Paget’s disease (pagetoid distribution of clear tumor cells in epidermis, with CAM5.2+ and CK7+ cells), Bowenoid papulosis (genital HPV infection with atypical keratinocytes and mitoses identical microscopically to Bowen’s disease), superficial spreading melanoma (pagetoid intraepidermal spread of atypical melanocytes in solitary elements or in nests, positive to S100, HMB45), and irritated seborrheic keratosis (absence of keratinocyte atypia, intense inflammation, and presence of mitoses due to the marked inflammation) [21] (Table 2).

In the current review, we also included keratoacanthoma as well since it is an epithelial neoplasm with a squamous differentiation. Keratoacanthoma is mainly divided into keratoacanthoma centrifugum marginatum (giant keratoacanthoma with striking centrifugal spread) and multiple keratoacanthomas [2,3]. Histopathologically, it is characterized by a symmetrical and circumscribed proliferation of keratinocytes, with a central horn plug, epidermis that extends over the tumor, and pale ground-glass-like keratinocytes with uniform differentiation [2,3]. The main histologic differentiation is the invasive SCC (Table 2). Keratoacanthoma can be considered a highly differentiated SCC, which only rarely escapes intrinsic tumor surveillance [2,3].

SCC is mainly divided in conventional, spindle-cells, verrucous, acantholytic, and lympho-epithelioma like SCC. The Broders classification is based on the degree of tumor differentiation [21]. More specifically, grade I includes tumors composed of <25% undifferentiated cells, grade II lesions with <50% undifferentiated, grade III lesions with <75% undifferentiated cells, and grade IV lesions with >75% undifferentiated cells [21].

**Figure 8:**
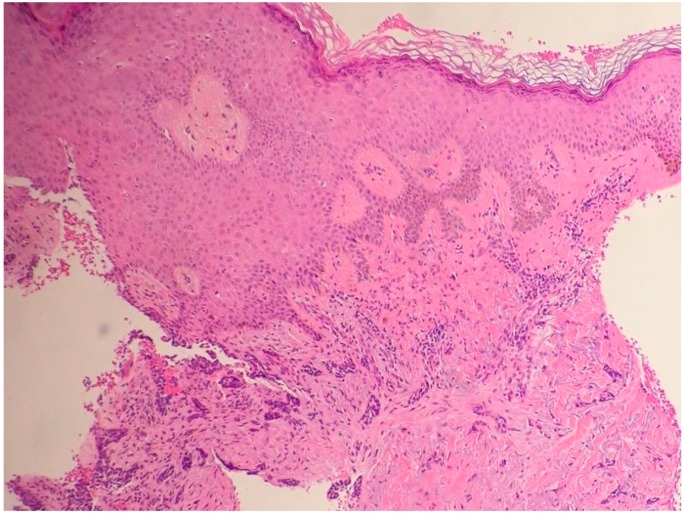
Desmoplastic squamous cell carcinoma (hematoxylin and eosin, 10×).

Conventional SCC is characterized by atypical cells in the dermis, showing enlarged and pleomorphic nuclei with atypical mitotic activity [21] (Table 2). Inflammation is usually present in ulcerated lesions and typically consists in lymphocytes, plasma cells, and neutrophils [1]. Keratinous pearls are typically present on the dermis, surrounded by nests of atypical cells and reduced stroma with lymphocytes [1]. The conventional SCC may also show desmoplastic features [21]. Desmoplastic SCC is characterized by aggregates of moderate or poorly differentiated keratinocytes surrounded by desmoplastic stroma, which by definition includes at least 30% of the tumor volume (Figure 8) [21]. This variant is associated with peri-neural invasion and higher metastatic spread [21].

Spindle cell SCC is associated with previous trauma or radiotherapy and it is the main variant associated with the Marjolin ulcer (post-traumatic ulcerated SCC) [21]. It is characterized by spindle cells that involve the dermis and intermingle with strands of collagen in a whorled fashion [29]. The surrounding stroma may be myxoid and pleomorphic giant cells may be appreciated, but desmoplasia should not represent more than 30% of tumor volume [21]. Mitotic activity is also present with atypical mitoses [26]. A possible subtype of SCC is pigmented SCC [21]. This subtype is rare and preferentially affects the oral and conjunctival mucosa in dark-skinned patients [19].

Acantholitic SCC (ASCC) is also known as adenoacanthoma [1,29,30,31,32]. ASCC is usually found on the head and neck in elderly people, above all in post-radiotherapy sites [21]. ASCC is characterized by an adenoid pattern, with a lot of dyskeratotic cells [1,2,3,33]. ASCC is similar to eccrine neoplasms, but it is PAS, negative for carcinoembryonic antigen and positive for epithelial membrane antigen [19]. A variant of ASCC is the pseudo-vascular adenoid SCC, which shows less acantholysis and a preminent glandular appearance [1]. This tumor is characterized by pseudovascular lumen-like features with connection to the overlying epidermis and tumor cells that dissect through dermal collagen and around adnexal structures [1]. The pseudo-vascular adenoid SCC cells show also hobnail features [1]. Finally, papillary SCC is more frequent in elderly women and in immunosuppressed patients; it is characterized by red to tan features [33].

Adenosquamous carcinoma is an uncommon variant, characterized by the simultaneous presence of mixed glandular and squamous differentiation, with an aggressive clinical behavior. Histologically, it is characterized by the presence of features of SCC with intercellular bridging, keratin pearl formation, parakeratotic differentiation, and an adenomatous component characterized by the presence of intracytoplasmatic mucin, although in the presence of true glandular formation, the presence of intra-cytoplasmatic mucin is not required to reach a diagnosis [34] (Table 2).

Finally, verrucous carcinoma of the skin and mucosa is a well-differentiated SCC, which when present in the genito-anal region is also known as the Buschke-Löwenstein tumor [35]. Histologically, it is characterized by exophytic squamous proliferation with marked papillomatosis and low atypia and the presence of koilocyte-like changes, central collection of neutrophils may be also present [35]. There is a lot of confusion regarding the terminology of this rare tumor; it was variously described as giant condyloma acuminate, squamous papillomatosis, condyloma acuminata with malignant transformation, and well-differentiated squamous cell carcinoma. However, verrucous carcinoma presents as a distinct entity with exo-endophytic growth pattern (in contrast to condyloma accuminata) of squamous cells, showing mild atypia with pushing margins (in contrast to the invasive character of well-differentiated squamous carcinoma) [30]. Finally, epithelioma cuniculatum is a rare variant of SCC of the foot. Histologically, it is characterized hyperkeratosis; acanthosis with an undulating, densely keratinized, well differentiated squamous epithelium, deeply penetrating the soft tissues; frequently separated by sinuses filled with inflammatory cells. Its pathogenesis is unknown, although it may be preceded by a plantar wart [36].

Generally, the main histological differential diagnoses of SCC are metastasis from internal squamous cell carcinoma (importance of the personal medical history of the patient, nodular proliferation without connection to epidermis, immunohistochemical evaluation), adnexal carcinoma (adnexal carcinoma may have squamous differentiation, but does not show connection with the epidermis and highlight adnexal features), keratoacanthoma (a highly differentiated SCC), and inverted follicular keratosis (sharply circumscribed endophytic verrucous proliferation with prominent squamous features) [2,3] (Table 2). 

## Figures and Tables

**Table 1:** Main histological differential diagnoses of basal cell carcinoma (BCC).

Differential Diagnosis	Pathological Features
*Tricoblastoma*	Absence of cleft, rudimentary hair germs, papillary mesenchymal bodies.
*ADC*	Lack of basaloid cells disposed in peripheral palisades; adenoid-cystic lesion without connection to the epidermis; absence of artefactual clefts
*MAC*	Bland keratinocytes, keratin cysts, ductal differentiation CEA+, EMA+ and BerEp4-
Tricoepithelioma *	Rims of collagen bundles, calcification, follicular/sebaceous/infundibular differentiation and cut artefacts. Cytokeratin (CK)20+, p75+, Pleckstrin homology-like domain family A member 1 + (PHLDA1+), common acute lymphoblastic leukemiaantigen + (CD10+) in tumor stroma, CK 6-, Ki-67– and Androgen Rceptor - (AR-)
*MCC*	Cells arranged in a diffuse, trabecular and/or nested pattern, involving also the subcutis. Mouse Anti-Cytokeratin (CAM) 5.2+, CK20+, S100-, human leukocyte common antigen – ( LCA-), thyroid transcription factor 1- (TTF1-)

ADC: adenoid cystic carcinoma; MAC: microcystic adnexal carcinoma; MCC: Merkel cell carcinoma; * above all desmoplastic tricoepithelioma.

**Table 2:** Main histological features and differential diagnoses of non-melanoma skin cancers with squamous differentiation.

Differential Diagnosis	Clinico-Pathological Features
*AK*	Atypical keratinocytes confined on basal layer.
*Bowen*	Atypical keratinocytes at every layer of epidermis.
*KA*	Symmetrical and circumscribed proliferation of keratinocytes, with central horn plug, with epidermis that extends over the tumor. Highly differentiated SCC.
*Invasive SCC*	Atypical and pleomorphic keratinocytes, involving the dermis and the sub-cutis with a potential metastatic spread.
*QE*	As Bowen, but in the mucosa.
*ACs*	Squamous differentiation, but does not show connection with the epidermis and highlights adnexal features.
*AD SCC*	Mixed glandular and squamous differentiation.
VSCC *	Exophytic squamous proliferation with marked papillomatosis and low atypia and the presence of koilocyte-like changes
*EC*	SCC of the foot. Histologically is characterized hyperkeratosis, acanthosis with an undulating, densely keratinized, well differentiated squamous epithelium, deeply penetrating the soft tissues.
*IFK*	Sharply circumscribed endophytic verrucous proliferation with prominent squamous features.
*SK*	Acanthosis, absence of atypia, pseudo-horn cysts, in inflamed lesions, mitoses may be present.
*BP*	Atypical keratinocytes and mitoses. Histology similar to Bowen’s disease.
*Metastasis*	Personal medical history of the patient, nodular proliferation without connection to epidermis, immunohistochemical evaluation.

AK: actinic keratosis; KA: keratoacanthoma; SCC: squamous cell carcinoma; QE: Queyrat’s erytroplasia; AC: adnexal carcinomas; IFK: inverted follicular keratosis; SK: seborrheic keratosis; BP: Bowenoid papulosis; AD SCC: adenosquamous carcinoma; VSCC: Verrucous squamous cell carcinoma. * When present in ano-genital region is also known with the term of Buschke–Löwenstein tumor; EC: epithelioma cuniculatum.
